# A Computational Design Framework for Efficient, Fabrication Error-Tolerant, Planar THz Diffractive Optical Elements

**DOI:** 10.1038/s41598-019-42243-5

**Published:** 2019-04-09

**Authors:** Sourangsu Banerji, Berardi Sensale-Rodriguez

**Affiliations:** Department of Electrical and Computer Engineering, University of Utah, Salt Lake City, UT 84112 USA

## Abstract

We demonstrate ultra-thin (1.5-3λ_0_), fabrication-error tolerant efficient diffractive terahertz (THz) optical elements designed using a computer-aided optimization-based search algorithm. The basic operation of these components is modeled using scalar diffraction of electromagnetic waves through a pixelated multi-level 3D-printed polymer structure. Through the proposed design framework, we demonstrate the design of various ultrathin planar THz optical elements, namely (*i*) a high Numerical Aperture (N.A.), broadband aberration rectified spherical lens (0.1 THz–0.3 THz), (*ii*) a spectral splitter (0.3 THz–0.6 THz) and (*iii*) an on-axis broadband transmissive hologram (0.3 THz–0.5 THz). Such an all-dielectric computational design-based approach is advantageous against metallic or dielectric metasurfaces from the perspective that it incorporates all the inherent structural advantages associated with a scalar diffraction based approach, such as (*i*) ease of modeling, (*ii*) substrate-less facile manufacturing, (*iii*) planar geometry, (*iv*) high efficiency along with *(v)* broadband operation, (*vi*) area scalability and (*vii*) fabrication error-tolerance. With scalability and error tolerance being two major bottlenecks of previous design strategies. This work is therefore, a significant step towards the design of THz optical elements by bridging the gap between structural and computational design i.e. through a hybrid design-based approach enabling considerably less computational resources than the previous state of the art. Furthermore, the approach used herein can be expanded to a myriad of optical elements at any wavelength regime.

## Introduction

Diffractive optics as a field has seen a lot of progress in the past decade^1^. The primary reasons for its success can be attributed to its simplicity in modelling, thin-geometry, relatively efficient structures and low-cost manufacturability (use of common 3D printable materials)^1,2^. In the literature, there are several reports on the design and manufacture of THz diffractive optical elements (DOEs) as compact and planar systems^3–9^. However, the primary disadvantages associated with DOEs are many. Firstly, the discontinuities of the wrapped phase typically introduce a shadow, which guides the incident beam into un-wanted orders^2,10^. Secondly, at other illumination frequencies apart from the blazing frequency (wavelength), the efficiency considerably decreases^2,11,12^. Thirdly, scalability is a major concern since the optical elements are either too small or too big for effective system integration^2,11^. Finally, current DOEs are not much tolerant to fabrication errors due to it stringent broadband phase matching condition^2,11,13^. These issues have greatly prevented a massive market rollout of DOEs in many applications. However, research efforts in the recent past from various groups^6–9^ have been addressing these problems. For instance, the first problem was addressed by utilizing a 2π phase jump to reduce the thickness of the elements to the wavelength scale with the usage of large refractive index materials^14,15^. The second problem was later addressed with the adoption of thick DOEs; since the basic theory for any kind of wavefront shaping is still based on phase accumulation along the optical path^7^. Still a few questions do persist like “*Are there other mechanism of phase and/or amplitude modulation that makes it possible to reduce the thickness of the optical elements for such broadband operation? How do we address the scalability and error tolerance issue? What are the alternatives to those?*” We do not deny the fact that researchers still have not fully addressed these questions. In fact, some progress in these directions have already been made by incorporating various hybrid design based computational frameworks^7,11,16–36^. Various optimal search strategies like direct search methods (which includes the most commonly used variant i.e. Gerchberg–Saxton algorithm)^16–20,22,23,27,33^, iterative fourier transform algorithms (IFTA)^21,24,25^, genetic algorithms^25,26,36^ and Monte Carlo optimization^34,35^ have already been implemented. In addition, lesser-known simulation strategies like angular spectrum method^28^ or even the point-to-point method^29^ have also been employed. These computational frameworks are rather slow with a huge computational resource burden^16,20,25,33^. Moreover, scalability remains an issue even with such algorithms because with large number of elements the computational problem approaches a Nondeterministic Polynomial (N.P.) time hardness^37^. In this context, as per our knowledge, there is still a scarcity of work in which researchers have employed a systematic framework to design DOEs at THz wavelengths.

In this work, our primary objective is to introduce a systematic design framework to design a myriad of arbitrary THz optical elements by adopting and modifying one of the most widely used algorithms i.e. the Direct Binary Search (DBS) algorithm;^16–20,22,23^ thereby employing a Gradient Descent Assisted Binary Search (GDABS) technique. We demonstrate our approach with the help of three different design examples of varying design complexity. In the first design example of a high N.A. lens, we go through the entire rigorous cycle of design, fabrication, and measurement with an additional verification step with FDTD simulations. We also perform an error sensitivity analysis of our designs. The last two design examples, i.e. a spectral splitter and a hologram, just touch upon the design of the THz optical element to keep the discussion of this paper brief and general. We believe that a widespread adoption of such hybrid design based computational approaches is truly necessary to design the next generation of THz optical elements as well as pave the way towards the adoption of more advanced computational design alternatives for example machine learning.

## Results and Discussion

Our approach relies on the careful modelling of scalar diffraction, where the diffraction pattern at a specific distance is determined from the phase accumulated by the THz waves transmitting from the diffractive structures’ surface topography in addition to the phase accumulated in air. For designing broadband DOEs, this is a very stringent condition because multiple frequencies must now diffract from the diffractive element (DE) and interfere constructively at the observation plane. Theoretically speaking, a phase condition providing constructive interference at the focus or observation plane, and destructive interference elsewhere, is not possible; hence, 100% efficiency is unattainable^16^. Based on this theoretical understanding of the physics of scalar diffraction we proceed to incorporate such in our computational design.

### Optimization based search algorithm

To solve this nonlinear search problem, a variation of the DBS algorithm^16–20,22,23^ has been studied and implemented. The optimization based search algorithm operates on the assumption that the solution for the specified design problem statement already exists i.e. a case of multiple local minima within the given search space which is at the heart of a non-convex optimization search problem. The following convexity in the problem makes the search much easier than the general case since it gives two important insights. Firstly, the availability of multiple local minima in the search space and secondly, first order conditions are sufficient conditions for optimality. This reduces the complexity to a local search optimization problem.

**Figure 1:**
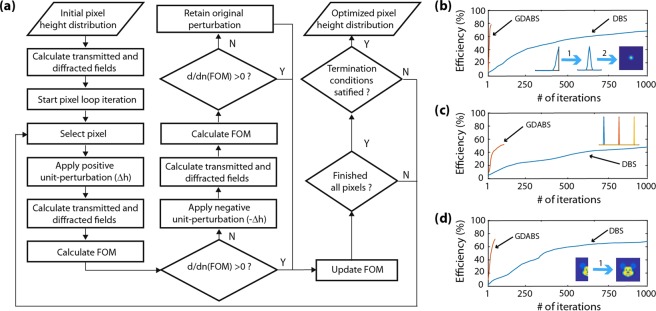
(**a**) Flow diagram of the Gradient Descent Assisted Binary Search (GDABS) algorithm. The right panels depict convergence plots of efficiency with number of iterations for (**b**) high N.A. broadband aberration rectified spherical lens with a two-fold symmetry (i.e. left to right flip symmetry and rotational symmetry) condition, (**c**) spectral splitter with no symmetry condition and (**d**) an on-axis broadband transmissive hologram with one-fold symmetry (i.e. left to right flip symmetry) condition.

Figure 1(a) depicts the flow diagram of the proposed algorithm. Once the initial pixel height profile is set, the algorithm starts by traversing each pixel in the DOE and performing a perturbation by increasing the height by ∆h. Next, the transmitted field, the diffracted field and the Figure of Merit (FoM) function is calculated. If the new FoM value is lower than the previously stored FoM value, the perturbation is accepted and the FoM is updated. Otherwise, the height of the individual pixel is now decreased by ∆h and again the transmitted field, the diffracted field and the Figure of Merit (FoM) function is calculated. If the new FoM value is lower, this new perturbation is accepted while concurrently updating the FoM value. Else, the perturbation is rejected overall and the pixel is returned back turns to its original height value while retaining the value of the previous FoM. The process moves forward by addressing every pixel in a sequential manner until all the pixels within a given iteration had been traversed. In simpler terms, the algorithm integrates a gradient descent based search routine, where the FoM of subsequent sub-iterations is compared to ensure a favorable gradient towards convergence. When the gradient became zero across an entire subsequent iteration, the termination condition is satisfied and the optimization process is terminated. An important point to note here is that many other termination strategies could also be implemented. Furthermore, imposing symmetry constraints on the initial pixel height profile can further reduce the computational burden. The next sub-section elucidates on this. Unlike the conventional perturbation-based iterative DBS technique employed in previous works^16–20,22,23^ integration of the gradient descent assisted search routine benefits with less computation time. Moreover, the gradient descent ensures that the optimized pixel height distribution is indeed the first local minima that we obtain when searching the solution space.

### Symmetry and convergence

The significance of a suitable initial pixel height profile is quite important to guarantee a fast and efficient solution. In this work, a more practical evaluation metric like the efficiency was taken so as to illustrate the algorithm convergence in contrast to the FoM metric, which was used during the optimization. For the first two design examples (high N.A. lens and spectral splitter), the efficiency of the predicted designs was defined as the ratio of the energy contained with the central lobe of the obtained gaussian profile to the total energy contained within the aperture area at the observation plane^17^. A slightly different efficiency metric was used for the third design example (hologram) where efficiency was calculated as the power inside the outline of the target image divided by the total power inside the aperture of the hologram^22^.

Figure 1(b–d) depicts the convergence of the algorithm (as computed efficiency vs. number of iterations) for three different design examples considered in this work. In all the three cases, the DBS algorithm was terminated at 1000 iterations since the convergence of efficiency was not observed to improve beyond 10% after that point. A general observation was that the convergence in the case of the GDABS algorithm is at least 10-100X times faster than that of the conventional DBS algorithm for the same starting pixel height profile in all the cases, irrespective of whether symmetry was considered or not. Furthermore, across all the runs of the GDABS algorithm itself, it was also observed that increasing the number of symmetry constraints led to a faster convergence to a desired solution. This is intuitive and self-explanatory. However, it makes way for the claim that the optimization is consistent across the various constraints imposed over it.

### Design examples

We now discuss in detail the design of three distinct THz optical elements using our optimization based search algorithm as described in the previous section.

### High N.A. broadband aberration rectified spherical lens

**Figure 2:**
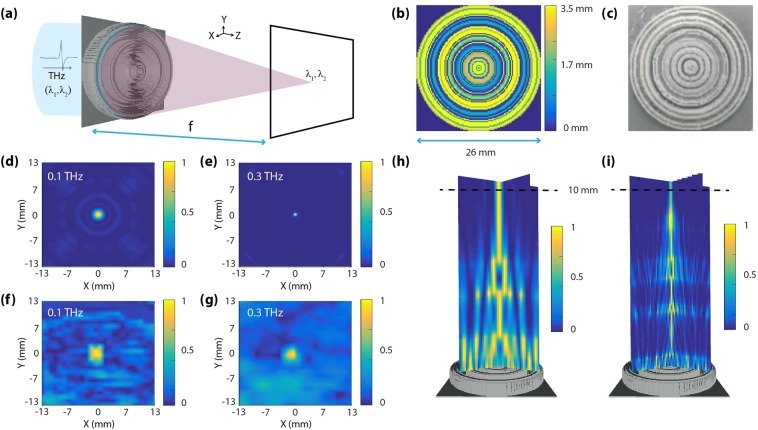
(**a**) Schematic of the high N.A. broadband aberration rectified spherical lens with a focal length f = 10 mm under broadband illumination (λ_1_ = 3 mm [0.1 THz] and λ_2_ = 1 mm [0.3 THz]). (**b**) Pixel height distribution for the spherical lens with a maximum height of 3.5 mm. The dimensions (length and width) of the designed lens was 26 mm. The pixelation is along both *x* and *y* directions. (**c**) Optical image of a fabricated spherical lens using a common 3D printer. PLA was the material used to print the structure. The bottom left panels depict the (**d**,**e**) scalar predication based semi-analytic and (**f**,**g**) measured on-axis point spread functions (PSFs) for 0.1 THz and 0.3 THz. The bottom right panels depict the (**h**,**i**) z-propagation scalar plot for the designed spherical lens.

The most basic THz optical element i.e. a high N.A. broadband aberration rectified spherical lens (Fig. 2(a)) was designed using our optimization based search algorithm. The rationale behind the chosen design parameters was guided by the limitations of the current state of the art 3D printers. The spherical lens was designed to be 26 mm in both length and width. The surface topography consisted of multilevel pixels having a maximum thickness h_max_ = 3500 μm, minimum thickness h_min_ = 100 μm, and Δh = 100 μm height level step; which sets the number of distinct height levels (P) to P = 35. The pixels have a width w = 200 μm; this sets the number of pixels (N) to N = 130 × 130 = 16900. A schematic depicting the relevant geometric dimensions of the lens is depicted in Fig. 2(b).

In terms of optimization parameters, the most critical parameter is the Figure of Merit (FoM) function. In this design and throughout this work, it was chosen in a way to match the diffracted field pattern in an ideal situation. From a mathematical perspective, the FoM metric reveals how closely the current predicted semi-analytic solution approximates the ideal diffracted field pattern. Likewise, from a physical perspective, the same FoM metric indicates how “*efficient*” the solution is. The second most critical parameter is the target function. In this case, it was defined as a wavelength specific diffraction limited gaussian profile with full-width-at-half-maximum (FWHM) determined by the desired numerical aperture. The details about the mathematical formulation of the FoM metric and the target function are provided in the Supplementary Information. Furthermore, a two-fold symmetry condition was also incorporated to speed up the computation within the optimization algorithm.

A conventional inexpensive 3D printing technique was employed to fabricate the lens^7,9,38–42^. The lens was then 3D printed with PLA (Poly (lactic) acid) and experimentally tested using a continuous wave THz imaging setup; details of which are provided are in the Supplementary Information. Figure 2(c) shows an optical image of the fabricated spherical lens. PLA was taken up as the material of choice primarily due to its widespread availability and ease of printing and coupled with the fact; that its absorption coefficient (k) is nearly ~0 across the entire bandwidth in which the lens was designed to operate. Despite this, during the design phase, both the refractive index (n) as well as the absorption coefficient (k) values were incorporated in the optimization algorithm.

Figure 2(d–g) depicts the scalar prediction based semi-analytic and measured on-axis PSFs for both 0.1 THz and 0.3 THz respectively. The z-propagation scalar plot as shown in Fig. 2(h,i) for both the designed frequencies portrays how the incident electromagnetic THz radiation traverses the optical path length in air before coming into focus at 10 mm. The semi-analytic PSFs of Fig. 2(d,e) show excellent achromatic focusing. Although the semi-analytic PSFs could not be exactly replicated due to the resolution and limited dynamic range of the imaging setup available in our laboratory, a good qualitative focusing at the focal plane was observed. At this point, a cross-validation with a full wave FDTD solver was carried out to check the validity of our scalar prediction-based model. The results of our semi-analytic PSFs were further corroborated with the FDTD PSFs as plotted in Supplementary Information (Fig. S2(a–d)). Another important point, which was also validated from the FDTD simulations, was the fact; that the designed lens was indeed polarization insensitive (in consistent with a scalar prediction based model). The z-propagation FDTD plots are also provided in Supplementary Information (Fig. S2(e–h)). The FWHM as calculated from the semi-analytic PSFs was 1.940 mm for 0.1 THs whereas the FDTD simulations predicted a value of 1.962 mm (under s-polarization) and 1.964 mm (under p-polarization). In case of 0.3 THz, the FWHM from semi-analytic PSFs was 0.710 mm; in contrast to a value of 0.747 mm (under s-polarization) and 0.744 mm (under p-polarization). The plots for the FWHMs are provided in Supplementary Information (Fig. S3). For both 0.1 THz and 0.3 THz, the FWHM values were a little wider than the theoretical diffraction limit of 1.9 mm and 0.63 mm respectively. Therefore, the simulations predict a diffraction-limited focusing behavior. More details about the FDTD simulations and results are provided in Supplementary Information.

**Figure 3:**
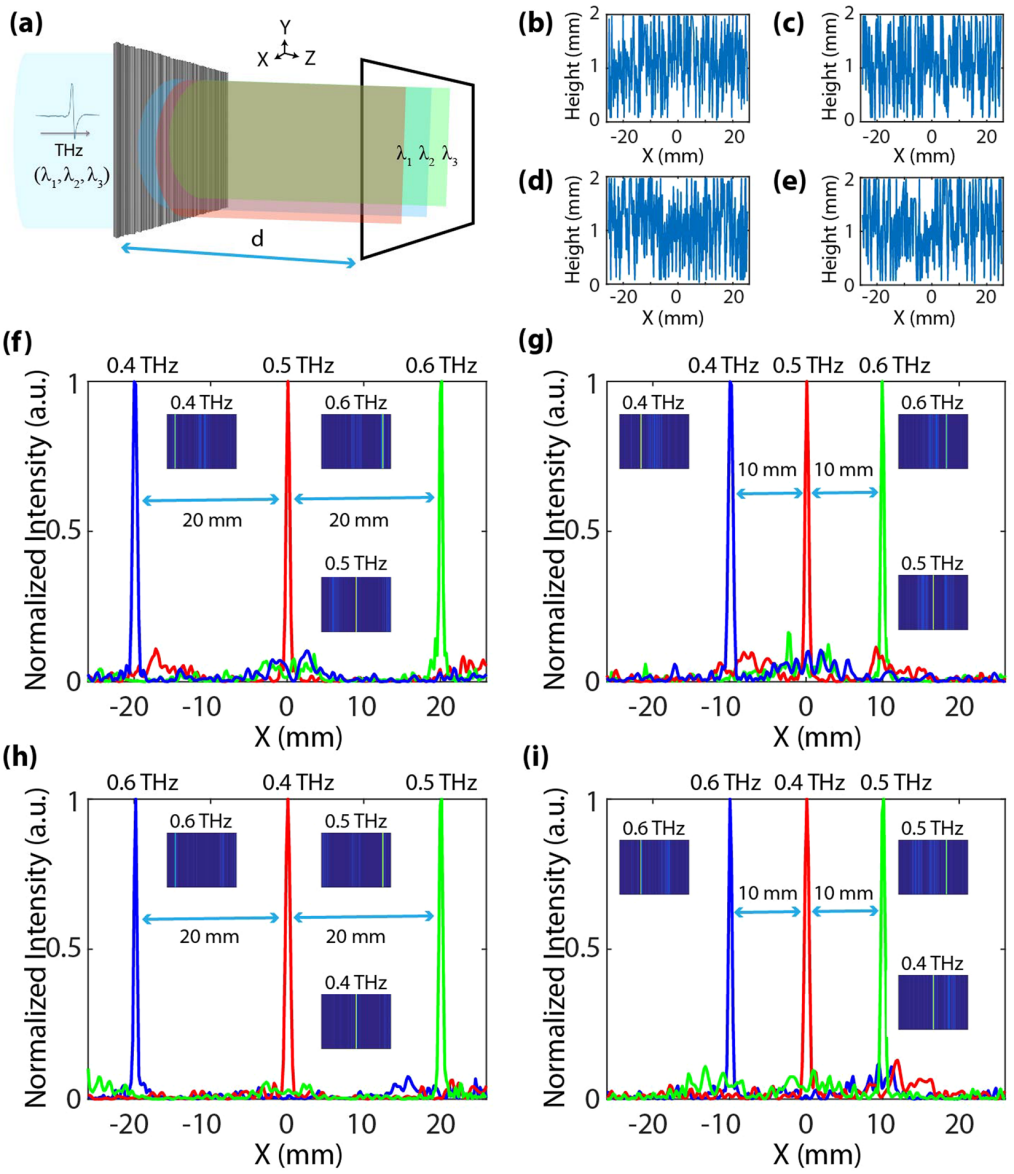
**(a)** Schematic of the spectral splitter with a splitting distance d = 50 mm under broadband illumination (λ_1_ = 0.75 mm [0.4 THz], λ_2_ = 0.6 mm [0.5 THz], and λ_3_ = 0.5 mm [0.6 THz]). The designed structure splits the incoming THz wave into a series of spatially separated lines at the pre-setted designed distance. (**b**–**e**) Pixel height distribution for the spectral splitter with a maximum pixel height of 2 mm. The dimensions (length and width) of the spectral spiltter was 52 mm. The pixelation is only along *x* direction. The bottom panels depict the designed spatial profile for splitter elements with uniform splitting at an inter-spatial distance of (**f**) 20 mm, and (**g**) 10 mm and also spectral splitters with an arbitrary splitting at a inter-spatial distance of (**h**) 20 mm, and (**i**) 10 mm. The insets give the corresponding on-axis PSFs plots for the respective THz frequencies.

Any differences in the designed and experimental results at this stage; could very well be attributed to several contributing factors apart from the design and measurement itself. Firstly, the non-uniform illumination of the diffractive structure across its entire surface topography (Supplementary Information (Fig. S7(b,c))). Secondly, misalignment of the optical center, despite the measurement setup being carefully configured to maintain the center of the beam on the optical axis is also quite possible. Thirdly, the aperture used to scan the focal plane had a diameter bigger than (Supplementary Information Fig. S2(a)) the size of a pixel of the spherical lens which could dampen the accuracy of the result. Finally, fabrication imperfections could also affect the performance. A section have been devoted to this later on to bring out some fruitful insights on the issue. However, in spite of such major shortcomings of our limited measurement facility, which compromise the accuracy of our experimental results, the measured focusing properties of the designed lens were in good qualitative agreement with our expectations.

### Spectral splitter

The second THz optical element designed using the optimization-based search algorithm is a spectral splitter, i.e. analogue of optical transmission gratings, where different THz frequencies could be diffracted and focused into a series of spatially separated lines at pre-defined locations on the observation plane (Fig. 3(a)). In terms of design parameters, the dimension of the splitter was 52 mm in both length and width; consisting of multilevel pixels having a maximum thickness h_max_ = 2000 μm, minimum thickness h_min_ = 100 μm, and Δh = 50 μm height level step; which sets the number of distinct height levels (P) to P = 40. The pixels have a width w = 200 μm; this sets the number of pixels (N) to N = 260. Under broadband illumination, the splitter was designed to split the incident radiation at a distance, d = 50 mm. In total, four different spectral splitters were designed to portray the robust and dynamic design capability of our computational framework. Figure 3(b–e) depicts the pixel height profile along with relevant geometric dimensions for all of the designed spectral splitters.

Speaking of optimization parameters, a similar FoM metric as used in the earlier case of a spherical lens was employed but with a minor modification; the target function was defined via dictating the intensity field generated at the observation plane by a certain range of frequencies. Detailed specifics about the FoM and the target function are provided in the Supplementary Information. No symmetry condition could be incorporated to speed up the computation due to the inherent asymmetric nature of the problem. PLA was again the material of choice when defining the optical constants in the design. A value of n = 1.357 and k = 0.051 at 0.4 THz, n = 1.360 and k = 0.067 at 0.5 THz and n = 1.367 and k = 0.097 at 0.6 THz were employed.

Figure 3(f,g) depicts the spectral maps of two spectral splitters, which were designed to split the incident THz radiation in a regular sequence across the observation plane at a pre-determined inter-spatial distance of 20 mm (Fig. 3(f)) and 10 mm (Fig. 3(g)) for the designed frequencies of 0.4 THz, 0.5 THz, and 0.6 THz. In contrast to this, a separate set of two spectral splitter designs were also designed to split the incident THz radiation in an arbitrary, i.e. non-monotonic, sequence across the observation plane at a pre-determined inter-spatial distance of 20 mm (Fig. 3(i)) and 10 mm (Fig. 3(j)) respectively for the same set of frequencies. A fifth design was also made (Supplementary Information Fig. S4) which depicts the condition of an arbitrary non-monotonic spectral split at non-uniform arbitrary distances on the observation plane. In all the cases, the spectral plots portray good spectral behaviors. However, an important observation can be made. The designs, which had a random non-monotonic split of the incident THz frequencies, did relatively better in contrast to their regular-sequence counterparts. The primary reason can be attributed to the fact that, under alike geometric conditions, the spectral resolution offered by the non-regular-sequence design tends to be higher than that by the design with regular spectral split due to the spectral correlation function. Spectral correlation function measures how similar the diffraction patterns are at two distinct wavelengths. In the design of a regular-sequence spectral splitter, the spatial-spectral map changes relatively smoothly, in contrast to that of a random design, which often experiences abrupt variations. Therefore, the correlation function of the random design becomes narrower, i.e. it becomes easier to distinguish between wavelengths. These observations are consistent with those of earlier works reported in the literature^18–20^. A few papers providing an in-depth analysis behind such behavior have also been published^18,20,43,44^.

### On-axis broadband transmissive hologram

**Figure 4:**
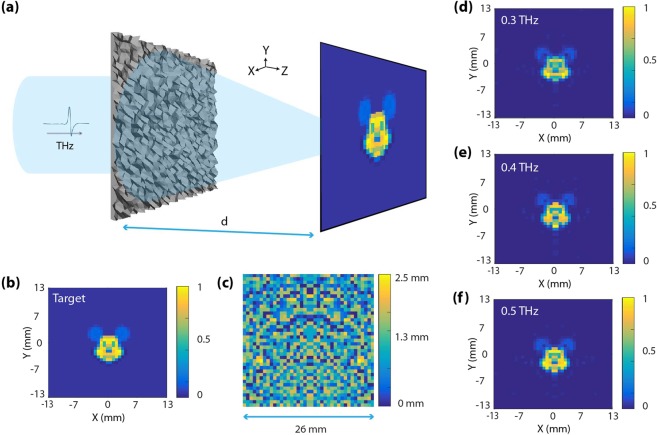
(**a**) Schematic of an on-axis broadband transmissive hologram with a operating distance d = 50 mm under broadband illumination (λ_1_ = 0.75 mm [0.4 THz], λ_2_ = 0.6 mm [0.5 THz], and λ_3_ = 0.5 mm [0.6 THz]). (**b**) The target image which was used during the optimization. (**c**) Pixel height distribution for the hologram with a maximum pixel height of 2.5 mm. The dimensions (length and width) of the hologram was 26 mm. The pixelation is along both *x* and *y* directions. The left panels depict the scalar predication based semi-analytic on-axis point spread functions (PSFs) for (**d**) 0.3 THz, **(e)** 0.4 THz and **(f)** 0.5 THz.

Using the GDABS algorithm, the last and most challenging THz optical element, which was designed, is an on-axis broadband transmissive hologram (Fig. 4(a)). Appropriate design parameters were chosen based on what could practically be fabricated and be used in specific applications. The square shaped hologram was designed with a side length equal to 26 mm. Similar to the previous designs, it also consisted of multilevel pixels having a maximum thickness h_max_ = 2500 μm, minimum thickness h_min_ = 100 μm, and Δh = 50 μm height level step; which sets the number of distinct height levels (P) to P = 50. The pixels have a width w = 650 μm; this sets the number of pixels (N) to N = 40 × 40 = 1600. Under broadband illumination, the hologram was designed to produce an image at a certain distance, d = 50 mm. Encoded within the hologram was a target image of a relatively complex scene i.e. the face of Mickey Mouse (Fig. 4(b)). A schematic depicting the multilevel pixel height profile along with the designed dimensions of the hologram is depicted in Fig. 4(c).

The only modification, which was made with respect to the previous two cases, is in the target function. A 2D matrix consisting of frequency encoded intensity values were inputted into the optimizer. Detailed information about the FoM and the target function are provided in the Supplementary Information. In this case, a one-fold flip (left to right) symmetry condition was incorporated to speed up the computation during the optimization process. The material, which was used during the design was again PLA.

The semi-analytic image (field intensity distribution at the observation plane) are shown in Fig. 4(d–f) for the respective pre-determined frequencies of 0.3 THz (Figs 4(d)), 0.4 THz (Fig. 4(e)) and 0.5 THz (Fig. 4(f)). Excellent color fidelity in terms of intensity distribution across the entire image plane is showcased from the plots of Fig. 4(d–f). The calculations of transmission efficiency for the designed hologram in this work followed a similar metric to those in ref.^45^ where it was defined as the ratio of the power transmitted through the hologram to that incident on the hologram. This metric was different from the FoM metric, which was used during the optimization process. The semi-analytic transmission efficiencies for this design were ~58%, ~71% and ~68% at 0.3, 0.4 and 0.5 THz, respectively. Finally, it is worth mentioning that using a much larger number of pixels as well as increasing the number of pixel heights, will greatly increase the reconstruction accuracy and will be the subject of further investigations.

### Parametric error tolerance analysis

**Figure 5:**
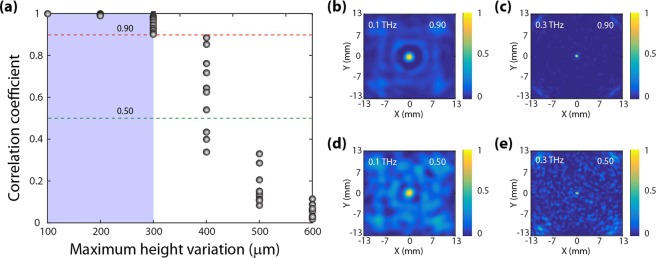
(**a**) Robustness of the designs to fabrication (standard 3D printing) errors. Random height variations of maximum value ΔE are introduced in the designs. Correlation coefficient is then computed between the diffraction patterns of the original and modified designs. The diffraction patterns are calculated on basis of the scalar predication based semi-analytic model. The correlation coefficient is then plotted versus ΔE for the designed lenses. The right panels depict the PSFs corresponding to 0.1 THz and 0.3 THz for (**b**,**c**) 0.90 correlation coefficient and **(d**,**e**) 0.50 correlation coefficient.

In order to provide an illustrative description of the tolerance of our designs to fabrication errors, without lack of generality, we chose to limit our analysis only to the lens design. As it is quite common with any fabricated structure, manufacturing errors could possibly affect the focusing performance of our designed lens. To quantify for such errors; we explicitly studied the tolerance of the designed diffractive structure to random pixel height variations. To account for such variations, the optimal design was altered by means of adding or subtracting a random value, Δe_ij_, to the height of each pixel (h_ij_). Δe_ij_ is such that 0 < |Δe_ij_| < ΔE, where ΔE was arbitrarily selected and represents the maximum fabrication error (maximum height variation). Diffraction patterns were then computed for the new structures containing these random variations in pixel height. The new diffraction pattern was then cross-correlated with the initial diffraction pattern, to calculate the cross-correlation coefficient (details are provided in the Supplementary Information). The cross-correlation coefficient was then employed as a metric revealing how close the new diffraction pattern was to the original pattern and hence was taken to be a measure of the fabrication error tolerance in our designed spherical lens. Our results are summarized in Fig. 5(a) where the lens was found to be strongly tolerant to random pixel height variations with maximum value ΔE ~300 μm. Figure 5(b–e) depict the corresponding PSFs for both 0.1 THz and 0.3 THz for two representative correlation coefficient values which is 0.90 (Fig. 5(b,c)) and 0.50 (Fig. 5(d,e)) respectively. Finally, we emphasize that many other manufacturing issues like uniformity of the material density, repeatability of fabrication, etc. could not be rigorously studied at this stage due to the unavailability of a dedicated 3D printing facility in our laboratory. The fabricated samples experimentally discussed in this manuscript were provided by a commercial vendor.

## Conclusions

We have discussed a computational design framework for the design of THz optical elements from the most basic responses (e.g. spherical lenses) to some of the most challenging ones (e.g. holograms). Based on the principles of scalar diffraction, our optimization-based search algorithm successfully integrates the theoretical concepts from the realms of diffractive optics and merges it with the principle algorithmic concepts of computer science and optimization. The subtle handling of the scalability in these THz optical elements was observed as the total design space was varied across all the three examples along with the usage of symmetry constraints. Irrespective of the constraints, the gradient descent based computational search routine, enabled up to 10-100X faster convergence than the traditional direct binary search technique. Furthermore, parametric error tolerance analysis elucidates the tolerance of such THz optical elements to fabrication errors. Barring the challenges associated with current 3D printing technology and the usage of better measurement facilities, our experimental demonstration of one such design example, i.e. a high N.A. spherical lens, evidences that the proposed design approach can be a significant step towards enabling new types of THz optical elements or even devices operating beyond the THz regime.

## Supplementary information

Supplementary Information
