# Supplementary material for: A Computational Design Framework for Efficient, Fabrication Error-Tolerant, Planar THz Diffractive Optical Elements
Source: Sci Rep. 2019 Apr 9;9:5801. doi: 10.1038/s41598-019-42243-5 (PMC6456492; doi:10.1038/s41598-019-42243-5)
Supplement: Supplementary file 1 — Supplementary Information [file 41598_2019_42243_MOESM1_ESM.pdf]

Supplementary Information for

"A Computational Design Framework for Efficient, Fabrication Error-Tolerant, Planar THz Diffractive Optical Elements"

Sourangsu Banerji[1] and Berardi Sensale-Rodriguez[1,*]

[1] Department of Electrical and Computer Engineering, University of Utah, Salt Lake City, UT 84112, USA

* berardi.sensale@utah.edu

1. Figure of Merit (FoM) function

The rationale behind choosing an appropriate figure of merit in our optimization based search algorithm was similar to the metric as followed in **[1].** The aim was to match the ideal diffracted field pattern, which would be formed based on the transmission field function $U_1(x', y', \lambda)$ (2D lens) in an ideal situation. The Figure of Merit (FoM) was defined in such a way that a lower value would indicate that the current semi-analytic solution closely approximates the ideal diffracted field pattern.

(i) High N.A. broadband aberration rectified spherical lens

For the high N.A. broadband aberration rectified spherical lens, the FoM of the algorithm has been defined as:

$$FoM = \frac{\sum_{i=1}^{N} \omega_i \theta_i}{N} - 10 \frac{\sum_{i=1}^{N} \omega_i \varphi_i}{N} \qquad (1)$$

The first and the second term in eqn. (1) describes the average weighted efficiency and the weighted normalized absolute difference over N frequency samples respectively. The weighting coefficient ω_i provides for balancing the contributions from different frequency samples. The frequency samples with higher weights follow (or "conform" to) their “target” quite closely, but those with lower weights do not; this is particularly useful in the design of polygrams **[2]**. A value of “1” is avoided to prevent over-fitting **[3]**. In this case, the value of $\omega_i = 0.99$ was used since we required the structure to have similar focusing across the entire range for all the frequency samples. When designing such diffractive structures for discrete frequencies, the value of N = 1 signifies that eqn. (1) can be written as,

$$FoM = \omega\theta - 10 * \omega\varphi \tag{2}$$

The physical importance of the scaling factor of 10 is equivalent to the -10 dB loss accounted for in any microwave and THz theoretical calculations. This provides for extra error tolerance in the designed diffractive THz structures. To be specific, a somewhat reasonable selection of the parameters is critical to achieve an optimal solution.

The expressions for efficiency θ_i and the normalized absolute difference φ_i over N wavelength samples for the spherical lens was written as,

$$\theta_i = \frac{\int_{x'_{min}}^{x'_{max}} \int_{y'_{min}}^{y'_{max}} I_i(x', y') T_i(x', y')\, dx'\, dy'}{\int_{x'_{min}}^{x'_{max}} I_i(\mathrm{x}', y')\, dx'\ dy'} \tag{3}$$

$$\theta = \frac{\int_{x'_{min}}^{x'_{max}} \int_{y'_{min}}^{y'_{max}} I(x', y') T(x', y')\, dx'\, dy'}{I(\mathrm{x}', y')} \tag{4}$$

and

$$\varphi_i = \frac{\int_{x'_{min}}^{x'_{max}} \int_{y'_{min}}^{y'_{max}} |Norm(I_i(\mathrm{x}', y')) - T_i(\mathrm{x}', y')|\, dx'\, dy'}{\int_{x'_{min}}^{x'_{max}} \int_{y'_{min}}^{y'_{max}} dx'\, dy'} \tag{5}$$

$$\varphi = \frac{\int_{x'_{min}}^{x'_{max}} \int_{y'_{min}}^{y'_{max}} |Norm\big(I(\mathrm{x}', y')\big) - T(\mathrm{x}', y')|\, dx'\, dy'}{\int_{x'_{min}}^{x'_{max}} \int_{y'_{min}}^{y'_{max}} dx'\, dy'} \tag{6}$$

where i = 1 denotes the case for discrete wavelength sample. Now the simulated electromagnetic intensity distribution term $I_i(x', y')$ and the defined target function T_i (x', y') are functions of both x' and y' at the observation plane for the ith wavelength sample. The limits of the integration range from x'_{min} to x'_{max} and y'_{min} to y'_{max} in both the x and y directions covering the entire area of the lens surface topography.

(ii) Spectral splitter

For the spectral splitter, the FoM of the algorithm has been defined as the same as in eqn. (1). In this case, the value of $\omega_i = 0.99$ for the frequency samples taken into consideration. However, the expressions for efficiency θ_i and the normalized absolute difference φ_i over *N* wavelength samples in this case for the 1D diffractive structure was modified as,

$$\theta_i = \frac{\int_{x'_{min}}^{x'_{max}} I_i(\mathrm{x}')T_i(\mathrm{x}')\, dx'}{\int_{x'_{min}}^{x'_{max}} I_i(\mathrm{x}')\, dx'} \tag{7}$$

$$\theta = \frac{\int_{x'_{min}}^{x'_{max}} I(\mathrm{x}')T(\mathrm{x}')\, dx'}{I(\mathrm{x}')} \tag{8}$$

and

$$\varphi_i = \frac{\int_{x'_{min}}^{x'_{max}} |Norm(I_i(\mathrm{x}')) - T_i(\mathrm{x}')|\, dx'}{\int_{x'_{min}}^{x'_{max}} dx'} \tag{9}$$

$$\varphi = \frac{\int_{x'_{min}}^{x'_{max}} |Norm\big(I(\mathrm{x}')\big) - T(\mathrm{x}')|\, dx'}{\int_{x'_{min}}^{x'_{max}} dx'} \tag{10}$$

where i = 1 denotes the case for discrete wavelengths. The term $I_i(x')$ denotes the simulated light intensity distribution function, and $T_i(x')$ is the designated target function for the ith wavelength sample. x'_{min} and x'_{max} are the limits of integration spanning from the leftmost to the rightmost of these diffractive THz structures.

(iii) On-axis broadband transmissive hologram

The FoM for the on-axis broadband transmissive hologram was kept similar to the FoM definition as used for the spherical lens since here too the main objective was to match the ideal pre-defined diffraction pattern at the observation plane. However, the value of ω_i as given in Eq. 1,

$$FoM = \frac{\sum_{i=1}^{N} \omega_i \theta_i}{N} - 10 \frac{\sum_{i=1}^{N} \omega_i \varphi_i}{N}$$

was taken to be different for the specific frequency samples. In this case, the best reconstruction of the hologram was desired at the center frequency of 0.4 THz within the entire broadband range of 0.3 THz – 0.5 THz for good contrast fidelity. Therefore, ω_i = 0.99 where "i" = 0.4 THz. A value of ω_i = 0.98 was taken for the rest of the frequency samples at 0.3 THz and 0.5 THz. Here also, a value of "1" was avoided to prevent overfitting during the optimization process.

Similar to the spherical lens case, here too, the expressions for efficiency θ_i and the normalized absolute difference φ_i over N wavelength samples was computed using Eq. 3 – Eq. 6. However, as will be discussed in the later section the defined target function T_i (x', y') were intensity matrices for the ith wavelength sample.

2. Target function

The appropriate target function was defined as a diffracted limited gaussian curve, whereby the diffracted field pattern at the observation plane should also resemble the same.

(i) High N.A. broadband aberration rectified spherical lens

For the high N.A. broadband aberration rectified spherical lens, under the first-order approximation, the focusing point-spread-function (PSF) was defined as a gaussian function centered at $\{\frac{(x'_{min}+x'_{max})}{2}, \frac{(y'_{min}+y'_{max})}{2}\}$ with full-width-at-half-maximum (FWHM), W_i determined by the far-field diffraction limit written as,

$$T_i(\mathrm{x}', y') = \exp\left\{-\frac{\left[x'-\left(\frac{(x'_{min}+x'_{max})}{2}\right)\right]^2+\left[y'-\left(\frac{(y'_{min}+y'_{max})}{2}\right)\right]^2}{\left(\frac{W_i}{2}\right)^2}\right\} \quad (11)$$

Here, W_i determined by the far-field diffraction limit. That is,

$$W_i = \frac{\lambda_i}{2NA} \quad (12)$$

$$NA = sin\left[\tan^{-1}\left(\frac{Lx}{2f}\right)\right] \quad (13)$$

Here, λ_i is the ith wavelength sample, NA denotes the numerical aperture of the lens, L_X and L_Y is the total length of the designed diffractive element in x direction and y direction; and f is the focal length. For discrete wavelengths i.e. i = 1, eqns. (11) becomes

$$T(\mathrm{x}', y') = \exp\left\{-\frac{\left[x'-\left(\frac{(x'_{min}+x'_{max})}{2}\right)\right]^2+\left[y'-\left(\frac{(y'_{min}+y'_{max})}{2}\right)\right]^2}{\left(\frac{W}{2}\right)^2}\right\} \quad (11.1)$$

where eqn. (8) is now given by

$$W = \frac{\lambda}{2NA} \tag{12.1}$$

which has a dependence on only the frequency sample for which the structures are designed for.

(ii) Spectral splitter

In case of the spectral splitter, under the first-order approximation, the point-spread-function (PSF) for such a 1D diffractive structure in the x direction only was defined as a gaussian function centered at $\frac{(x'_{max}+x'_{min})}{2}$ with full-width-at-half-maximum (FWHM), W_i determined by the far-field diffraction limit. That is,

$$T_i(\mathrm{x}') = \exp\left\{-\frac{\left[x' \mp \left(\frac{(x'_{rel\_pos}+x'_{max})}{2}\right)\right]^2}{\left(\frac{W_i}{2}\right)^2}\right\} \tag{13}$$

The expression for W_i remains the same as in eqn. (12)

Here, λ_i is the ith wavelength sample. The value "*rel_pos*" determined the sign in eqn. (13) which eventually determined the spatial location of the specific wavelength sample in the observation plane.

(iii) On-axis broadband transmissive hologram

For an on-axis broadband transmissive hologram, a relatively straightforward frequency encoded intensity matrix was defined as the target function and incorporated into the optimization process.

3. Cross-correlation function

We used the standard out of the box MATLAB cross coefficient function “xcorr” to extract out the cross-correlation coefficient in our post processing code.

The mathematical annotation for the same can be written as,

$$\rho_{XY}(\tau) = \frac{1}{\sigma_X \sigma_Y} E[(X_t - \mu_x)(Y_{t+\tau} - \mu_Y)] = \frac{1}{\sigma_X \sigma_Y} \gamma_{XY}(\tau)$$

where μ_X and σ_X are the mean and standard deviation of the process (X_t), which are constant over time due to stationarity; and similarly, for (Y_t), respectively. E[] indicates the expected value. That the cross-covariance and cross-correlation are independent of t is precisely the additional information (beyond being individually wide-sense stationary) conveyed by the requirement that (X_t, Y_t), are jointly wide-sense stationary.

4. FDTD simulations

The full wave FDTD simulations were carried out using Lumerical FDTD Solutions. The material properties (refractive index and absorption coefficient as a function of frequency) of PLA was imported into Lumerical directly as the structure's optical data. A ".lsf" script was written to replicate the lens geometry using the same dimensions which was specified during the optimization process as depicted in **Fig. S1 (a-c)**. An incident plane wave (type: diffracting [see link]) along the backward "z-axis" direction with both s (TE) and p (TM) polarization were used to illuminate the diffractive lens surface.

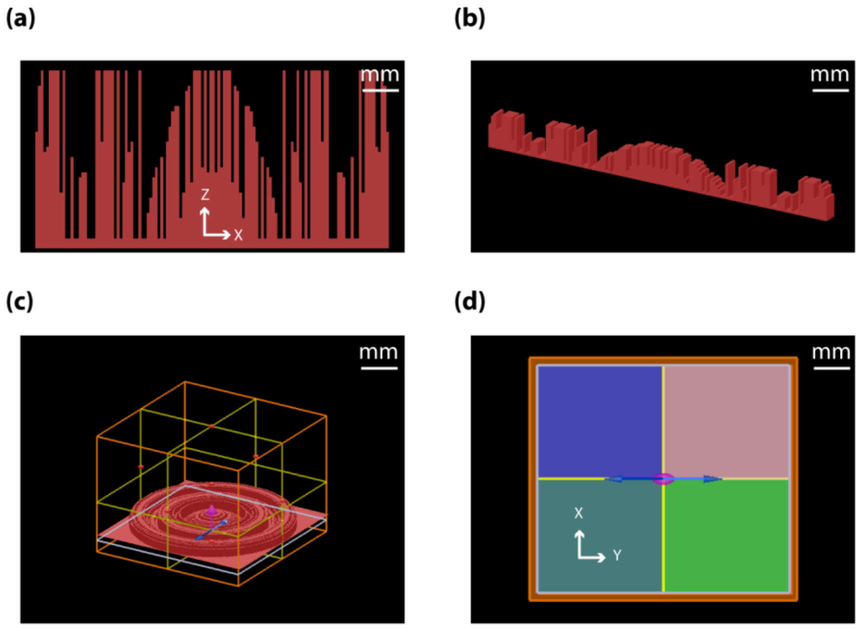

Figure S1 (a) 1D cross-section of the designed spherical lens cuss across the middle in Lumerical FDTD simulations **(b)** Perspective view of the 1D cross-section. **(c)** FDTD simulation setup of the spherical lens. **(d)** Boundary conditions imposed during the simulation (in case of TM polarization).

For the broadband THz excitation, the entire range or bandwidth of the pulse was defined (0.1THz - 0.3 THz). The entire FDTD simulation region was considered from the back surface of the spherical lens right up to 1.5 times the distance from the focal plane. A Perfectly Matched Layer (PML) boundary condition set up in the x-max, y-max and both z-min and z-max directions. As seen from **Fig. S1 (d)** that due to the inherent symmetry of the designed structure, the x-min boundary was set to "symmetric" and the y-min boundary was set to "anti-symmetric" (for TM polarization). Whereas the x-min boundary was set to "anti-symmetric" and the y-min boundary was set to "symmetric" (for TE polarization) which reduced the requirements by ¼ of the original simulation requirements in terms of both time and memory. We emphasize here that we tried to impose radial symmetry but could not as it has not yet been made available in the software [see link].

The default mesh was used to simulate the structures instead of a very fine mesh to avoid the huge computation time. The mesh accuracy was kept at "3" which has a good tradeoff for precision and accuracy versus the time and memory requirement. Field monitors placed at different planes above the lens and along the vertical plane to observe the field profiles of the propagating electromagnetic radiation.

(i) Point Spread Functions (PSFs) and beam propagation

The on-axis PSFs and the z-propagation of the THz beam for 0.1 THz and 0.3 THz under both s and p polarization as observed from the plots of **Fig. S2 (a-h)** corroborate the claim of polarization independent focusing with our designed spherical lens.

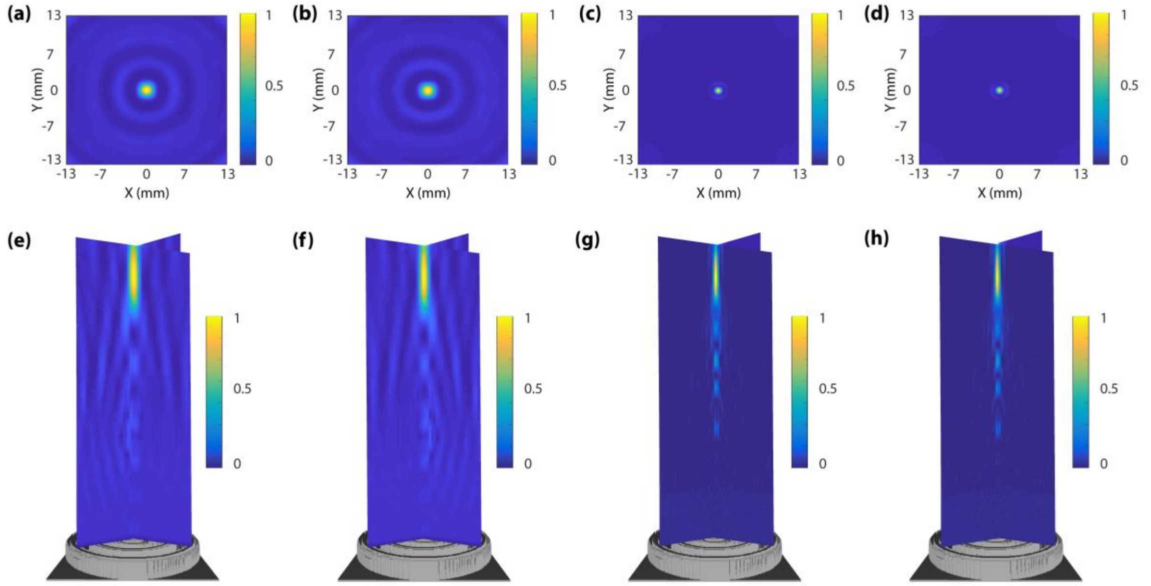

Figure S2 The top panel depicts the on-axis PSFs for under both s and p polarization for **(a-b)** 0.1 THz and **(c-d)** 0.3 THz. Subsequently the bottom panels describe the z-propagation FDTD plot for the designed spherical lens under both s and p polarization for **(e-f)** 0.1 THz and **(g-h)** 0.3 THz.

(ii) Full Width Half Maximum (FWHM)

The full width half maximum (FWHM) of our designed spherical lens for both the semi-analytic based prediction and the FDTD simulation are portrayed in **Fig. S3 (a)** 0.1 THz (with a theoretical diffraction limit value of 1.9 mm) and **Fig. S3 (b)** 0.3 THz (with a theoretical diffraction limit of 0.63 mm).

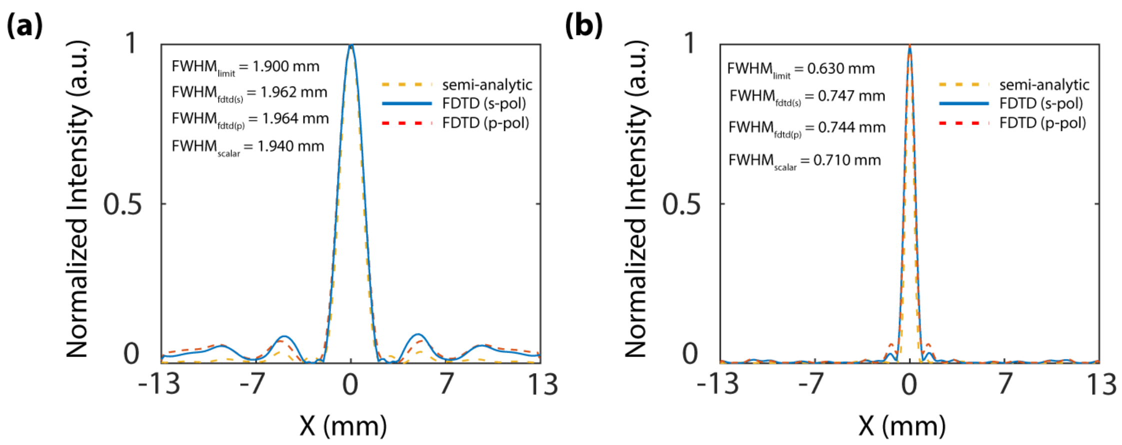

Figure S3 FWHM plot for **(a)** 0.1 THz and **(b)** 0.3 THz.

5. Spectral splitter (4-beam spectral splitter)

A spectral splitter element capable of splitting a broadband incoming THz radiation in an arbitrary sequence (0.4 THz, 0.6 THz, 0.3 THz and 0.5 THz) at a predefined arbitrary spatial location (both 10mm and 20 mm) on the observation plane was also designed as depicted in **Fig. S4** using the optimizer. The insets provide the corresponding on-axis PSFs plots for the respective THz frequencies.

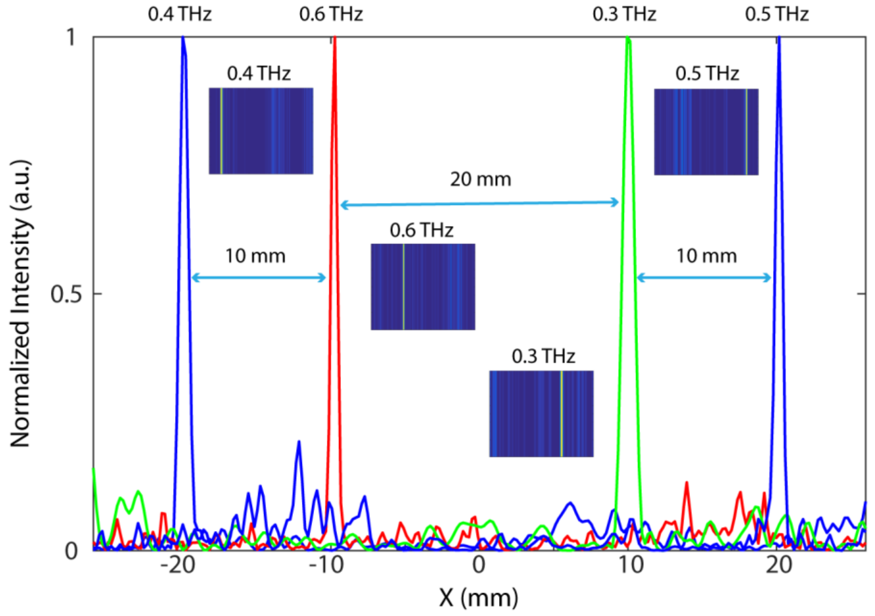

Figure S4 Spectral Splitter with arbitrary splitting at arbitrary inter-spatial distance.

6. Fabrication

The diffractive THz spherical lens was 3D printed by a commercial company called 3D Hubs (https://www.3dhubs.com/) with PLA (Poly (lactic) acid) **[4-5]**. A minimum layer height of 100 μm with a lateral resolution of 200 μm was 3D printed with appreciable accuracy.

(i) Material characterization

The refractive index (*n*) and the absorption co-efficient (*k*) of the 3D polymer material i.e. PLA was measured with the help of a THz-TDS system following the methods in **[6]**. The measurement result is plotted in **Fig. S5**. The refractive index of PLA is n = ~1.4 across the entire range from 0.15 THz – 0.65 THz lie in the lower bound of the typical reported values (n = ~1.53 ± 0.15) in the literature. The absorption coefficient k < 0.2 was within the range of reported values **[7-9]**.”

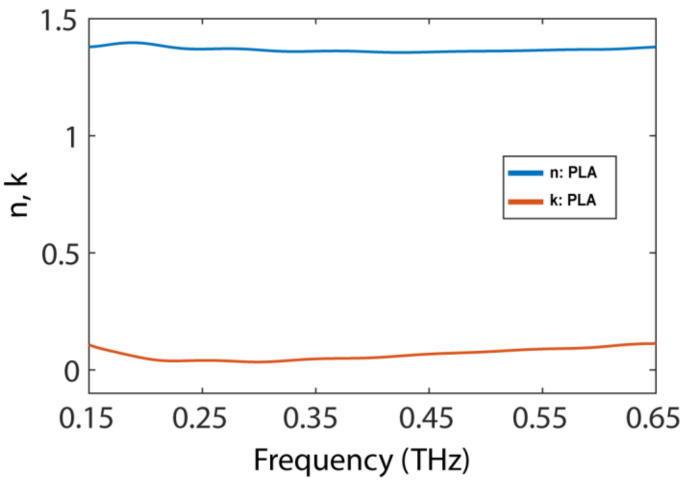

Figure S5 Refractive index and absorption coefficient of PLA material measured by THz TDS system

7. Measurement

For the high N.A. broadband aberration rectified spherical lens, a three-step approach were adopted to perform the measurement in our laboratory.

(i) General Procedure

The general procedure comprised of using a tunable diode laser-driven continuous wave terahertz (THz-CW) setup (Toptica Photonics). Here, tunable THz-CW radiation was generated by photo mixing of two spectrally separated fiber-coupled IR laser in InGaAs based photoconductive antennas, the radiation frequency was tuned by varying the diode temperature, hence emission wavelength of one of the source lasers. At receiver end, the THz radiation drives the optically excited carriers in the photoconductive antenna, generating current across the diode. The working of the THz-CW system is discussed in detail in **[10-11]**. For imaging the field profile through the designed lens, the lens was placed in the collimated region between the parabolic mirrors. A 0.25 mm diameter pinhole metal aperture was mounted, on a mechanical x-y translation stage, at the focal plane of the (10 mm distance) designed lens. The second parabolic mirror placed after this pin hole aperture, captures the traversing radiation, feeding the THz signal to the THz detector. The THz photocurrents were mapped over the focal plane by translating the pinhole aperture in x-y plane. The measured signal for the designed lens displayed qualitative focusing; which is a good first affirmation of our proposed design framework. In the next three sub-sections, we describe this in more details.

(ii) Process step-1

In the first step, the parabolic mirrors were aligned to maximize the THz radiation within the collimated region of the setup. **Fig. S6 (a)** gives the schematic of the measurement setup in our laboratory. **Fig. S6 (b-c)** depicts the maximum photocurrent (or intensity) levels obtained for 0.1 THz and 0.3 THz respectively. The collimated region was roughly 20 cm in our setup.

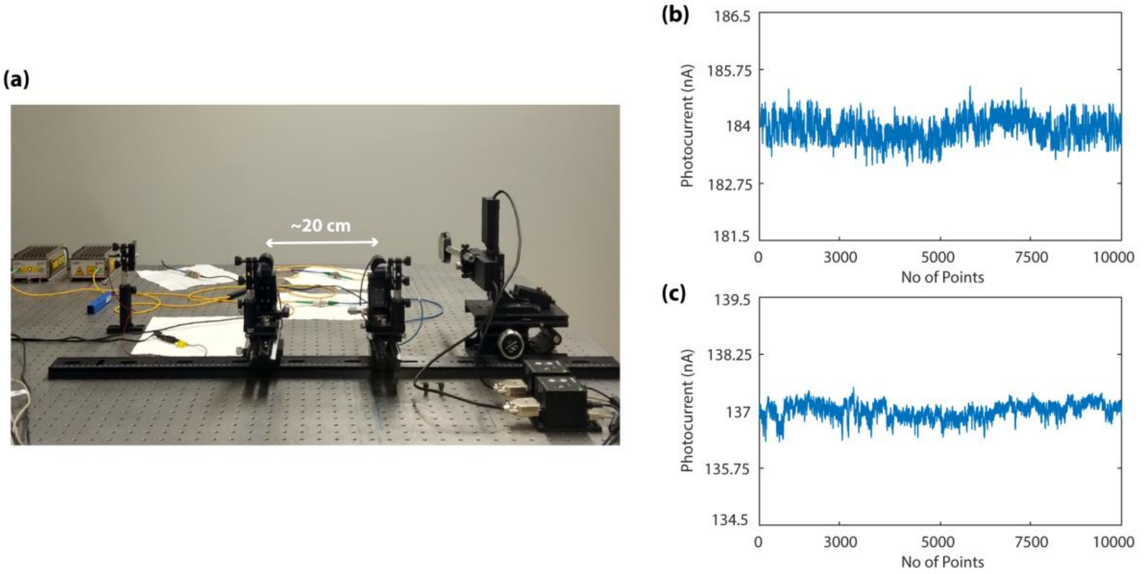

Figure S6 (a) Measurement setup for process step-1. Maximum photocurrent (or intensity) obtained in the setup for **(b)** 0.1 THz and **(c)** 0.3 THz.

(iii) Process step-2

In the next step, a metal aperture with 0.25 mm diameter was introduced within the collimated region. A scan was made to ascertain the amount of photocurrent that could be collected with this metal aperture without the lens in between as shown in **Fig. S7 (a)**. Each translation of the aperture was 0.2 mm (accounting for a total step count of 130 in

both x and y directions to cover 26 mm). To maximize the amount of photocurrent (or intensity), the metal aperture was kept only 1 cm away from the second parabolic mirror. **Fig. S7 (b-c)** depicts the un-normalized photocurrent (or intensity) levels obtained for both 0.1 THz and 0.3 THz respectively.

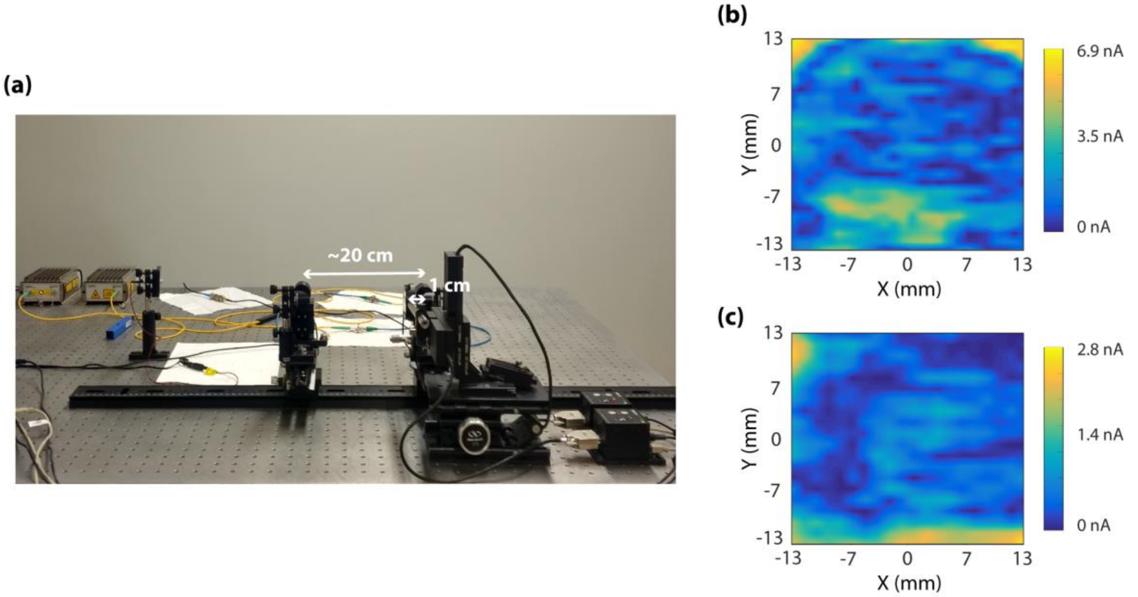

Figure S7 (a) Measurement setup for process step-2. Maximum photocurrent (or intensity) levels obtained in the setup for **(b)** 0.1 THz and **(c)** 0.3 THz.

(iv) Process step-3

Once it was made sure that no anomaly in the photocurrent (or intensity) levels were observed across the different frequencies. The designed spherical lens was placed in the collimated region approximately 1 cm (10 mm) away from the metal aperture as depicted in **Fig. S8 (a).** A scan was made using the metal aperture at the focal plane with the same

translation settings as in the previous step. **Fig. S8 (b-c)** depicts the un-normalized photocurrent (or intensity) levels obtained for both 0.1 THz and 0.3 THz correspondingly.

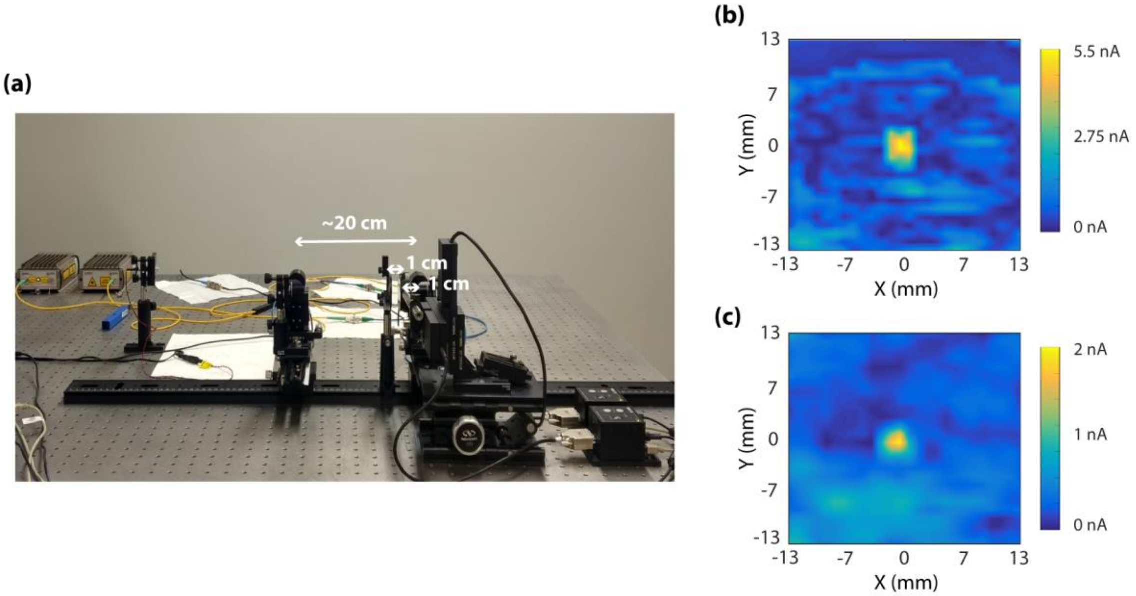

Figure S8 (a) Measurement setup for process step-3. Un-normalized photocurrent (or intensity) levels obtained in the setup for **(b)** 0.1 THz and **(c)** 0.3 THz at the focal plane.

References:

(1) Wang, P., Mohammad, N., and Menon, R. Chromatic-aberration-corrected diffractive lenses for ultra-broadband focusing. Sci. Rep., **2016**, 6, 21545.

(2) Meem, M. and Menon, R., Multi-level micro-optics enables broadband, multi-plane computer-generated holography. **2019**, arXiv preprint arXiv:1901.05943.

(3) Nguyen, T. and Sanner, S. Algorithms for direct 0–1 loss optimization in binary classification. In International Conference on Machine Learning **2013**, 1085-1093.

(4) Nampoothiri, K. M., Nair, N.R., and John, R.P. An overview of the recent developments in polylactide (PLA) research. Bio. Tech., **2010**, 101, 8493-8501.

(5) A. Podzorov, A., and Gallot, G. Low-loss polymers for terahertz applications. App. Opt. **2008**, 47, 3254–3257.

(6) Yahyapour, M., Vieweg, N., Roggenbuck, A., Rettich, F., Cojocari, O., and Deninger, A. A. Flexible phase-insensitive system for broadband CW-terahertz spectroscopy and imaging. IEEE Trans. Terahertz Sci. Technol., **2016**, 6, 670–673.

(7) Squires, A.D. and Lewis, R.A., Feasibility and Characterization of Common and Exotic Filaments for Use in 3D Printed Terahertz Devices. Journal of Infra., Milli., and Tera. Waves, **2018**, 39(7), pp.614-635.

(8) Squires, A.D., Constable, E. and Lewis, R.A., 3D printed terahertz diffraction gratings and lenses. Journal of Infra., Milli., and Tera. Waves, **2015**, 36, 72-80.

(9) Busch, S.F., Weidenbach, M., Fey, M., Schäfer, F., Probst, T. and Koch, M. Optical properties of 3D printable plastics in the THz regime and their application for 3D printed THz optics. Journal of Infrared, Millimeter, and Terahertz Waves, **2014**, 35(12), 993-997.

(10) Kim, J. Y., Song, H.J., Yaita, M., Hirata, A., and Ajito, K. CW-THz vector spectroscopy and imaging system based on 1.55-μm fiber-optics. Opt. Exp., **2014**, 22, 1735–1741.

(11) Naftaly, M., and Miles, R. E., Terahertz time-domain spectroscopy for material characterization, Proc. IEEE, **2007**, 95, 1658-1665.
